# A Microfluidic DNA Sensor Based on Three-Dimensional (3D) Hierarchical MoS_2_/Carbon Nanotube Nanocomposites

**DOI:** 10.3390/s16111911

**Published:** 2016-11-14

**Authors:** Dahou Yang, Mahnoush Tayebi, Yinxi Huang, Hui Ying Yang, Ye Ai

**Affiliations:** Pillar of Engineering Product Development, Singapore University of Technology and Design, Singapore 487372, Singapore; dahou_yang@mymail.sutd.edu.sg (D.Y.); mahnoush58@aut.ac.ir (M.T.); yinxi_huang@sutd.edu.sg (Y.H.); yanghuiying@sutd.edu.sg (H.Y.Y.)

**Keywords:** MoS_2_/MWCNT nanocomposites, DNA fluorometric detection, microfluidic biosensing

## Abstract

In this work, we present a novel microfluidic biosensor for sensitive fluorescence detection of DNA based on 3D architectural MoS_2_/multi-walled carbon nanotube (MWCNT) nanocomposites. The proposed platform exhibits a high sensitivity, selectivity, and stability with a visible manner and operation simplicity. The excellent fluorescence quenching stability of a MoS_2_/MWCNT aqueous solution coupled with microfluidics will greatly simplify experimental steps and reduce time for large-scale DNA detection.

## 1. Introduction

Over the last two decades, various nanomaterials including graphene oxide [1,2,3,4], Au nanoparticles [5,6,7], metal-organic framework (MOF) [8,9,10], carbon nanotubes [11,12], and MoS_2_ nanosheets [13,14,15,16,17,18,19] have been synthesized to develop nanomaterial-based biosensors for a variety of applications. In particular, there has been increasing interest in developing rapid, cost-effective, and sensitive methods for DNA detection because of its vital importance in molecular diagnostics, environmental monitoring, and anti-bioterrorism [3]. Among these nanomaterials for DNA detection, 2D MoS_2_, evolved as a fluorescence nanoquencher, has recently attracted extensive research efforts due to its unique properties [20,21,22]. Despite the advantages of MoS_2_, such as high quenching efficiency, easy synthesis at a large scale, and good dispersion in aqueous solutions [23], its quenching ability is not very stable and would be strongly affected by moisture and oxygen ubiquitous in the environment [24]. Furthermore, similar to the 2D graphene, the freshly synthesized MoS_2_ nanosheets have a tendency to aggregate in practical applications, which can greatly reduce the electrochemical active sites [25]. In our previous work [15], we found that the quenching ability of MoS_2_ aqueous solutions can substantially degrade within one day due to oxidization. It is vital to perform experiments immediately after preparation of the MoS_2_ aqueous solution, which is not convenient for a routine DNA test. The combination of MoS_2_ and other materials may overcome this deficiency. A MoS_2_/multi-walled carbon nanotube (MWCNT) composite as an electrode in a Li battery has shown a higher capacity and better cycling stability compared with pure MoS_2_ [26,27]. It was also reported that the thermal stability of the MoS_2_/MWCNT composites was better than MWCNT and MoS_2_ [28]. Huang et al. applied MoS_2_/MWCNT as an electrochemical biosensor that used graphene oxide as a redox marker while a thiol-tagged DNA probe was assembled on a MoS_2_/MWCNT and AuNP-modified electrode [28]. However, little attention has been paid to its application as a nanoquencher for DNA sensing. Here, to solve the degradation problem of pure MoS_2_ nanosheets for DNA detection, a novel 3D nanomaterial with MoS_2_ nanosheets grown on MWCNT is proposed in this work as a new fluorescence nanoquencher with high stability and sensitivity to detect single-stranded DNA (ssDNA) that can be adsorbed by van der Waals forces between MoS_2_ and nucleobases [13] and even helical wrapping to the MWCNT surface through п-stacking [29]. The novel MoS_2_/MWCNT composite is in a three dimensional (3D) hierarchical structure with 1D MWCNTs as back bones and the 2D MoS_2_ grown on the outer surface of MWCNTs with partially standing branch features [26], which provides a much larger material surface area for DNA sensing. The MoS_2_/MWCNT-based DNA sensing is performed in a microfluidic channel device that helps maintain uniform fluorescence intensity and thus ensures consistent fluorescence measurements in different locations. Using microfluidic channels can substantially reduce the sample volume needed for fluorescence measurement, typically less than 0.2 μL. This microfluidic chip has five identical channels, which offers a simple, rapid, portable, and high-throughput analysis method for screening of DNA.

## 2. Experimental Section

### 2.1. Reagents and Apparatus

Synthesized and purified DNA oligonucleotides were directly purchased from Integrated DNA Technologies Pte. Ltd. in Singapore. The sequences of all DNA oligonucleotides are shown in Table 1.

MWCNTs (L-MWCNTs-60100) (SEM image is shown as the supplementary information in Figure S1) were purchased from Shenzhen Nanotech Port Co., Ltd., Shenzhen, China. Other chemicals were purchased from Sigma-Aldrich Pte. Ltd., St. Louis, MO, USA. The deionized water used in all the experiments was purified using a Millipore filtration system. Fluorescence images were taken with a CCD camera (Leica, Model MC120HD, Wetzlar, Germany) on an inverted fluorescence microscope (Leica, Model DMIL LED). Fluorescence spectra were measured by Raman spectroscopy (WITec alpha300 R, WITec Pte. Ltd., Ulm, Germany) at the excitation wavelength of 532 nm. Laser spot was focused into a circle with a diameter of ~1.3 µm, and the fluid volume in the optical lightpath was 5.309 × 10^−8^ µL. All measurements were performed at room temperature in 1× phosphate buffered saline (PBS, pH 7.4), which contains 11.9 mM phosphatase, 2.7 mM potassium chloride, and 137 mM sodium chloride.

### 2.2. Synthesis of MoS_2_/MWCNT

MoS_2_/MWCNT nanocomposites were obtained using solvent thermal method [26], in which *N*,*N*-dimethylformamide (DMF) and (NH_4_)_2_MoS_4_ were used as the solvent and single reactant, respectively. In a typical experiment, briefly explained, 220 mg (NH_4_)_2_MoS_4_ powder and 100 mg MWCNTs were mixed first with the 30 mL solvent and sonicated to achieve uniform dispersion. Thereafter, an autoclave at 200 °C for 10 h was applied to form MoS_2_/MWCNT composites. After separation of the product by centrifugation (9391 g, 5 min), the purified MoS_2_/MWCNT composites were further washed up by DI water at least 5 times. Three different ratios of MoS_2_/MWCNT were synthesized by varying the Mo/Carbon ratio of 1:20, 1:10, and 1:5.

### 2.3. Optimization of Detection Conditions

The loading effect of MoS_2_ and MWCNT on the DNA detection was investigated by using three different MoS_2_/MWCNT ratios: 1:20, 1:10, and 1:5. A portion of 100 nM dye-labeled probe DNA was incubated for 10 min with different ratios of MoS_2_/MWCNT at room temperature. The concentrations of the different MoS_2_/MWCNT ratios are all 150 µg/mL. The fluorescence spectra were measured using Raman spectroscopy at the same location near the end of the microchannel. Similar procedures for the best performance ratio were also performed to investigate the effect of different concentrations of MoS_2_/MWCNT nanocomposites on the fluorescence quenching ability. Fluorescence spectra of pure PBS solution tested in the microchannel were used as the baseline, and results presented in this work were spectra minus this baseline. Error bars are obtained from at least six groups of data.

### 2.4. Fluorescent DNA Assays

Zigzag-shaped polydimethylsiloxane (PDMS) microchannels (Figure S2) were used as a platform for fluorescence measurement and imaging. The mixed samples were added into each channel in the device by a micro pipet in a volume of 0.2 µL and Raman spectroscopy was used to measure the fluorescence spectra at the end of the microchannels. In a typical DNA assay, the fluorescent probe P1 was hybridized with the targets in 1 × PBS (pH 7.4) for 10 min, after which the resulting solution was uniformly mixed with MoS_2_/MWCNT aqueous solution using a vortex mixer. All the concentrations mentioned in the following measurements are final concentrations in the mixture.

## 3. Results and Discussion

### 3.1. Working Mechanism

**Figure 1:**
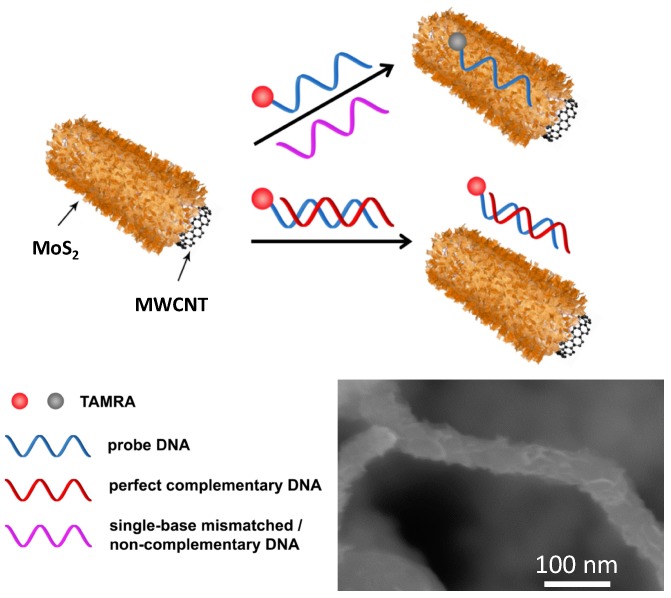
Schematic illustration of the MoS_2_/MWCNT nanocomposites-based fluorescence DNA sensing assay. Inset shows an SEM image of the MoS_2_/MWCNT nanocomposites.

As depicted in Figure 1, a probe DNA (P1) labeled with TAMRA is utilized to detect its perfect complementary DNA (T1) where TAMRA was excited at 532 nm and the emission wavelength was 580 nm. MoS_2_/MWCNT (SEM image is shown in Figure 1, inset) can adsorb a TAMRA-labeled single-stranded probe DNA (P1) via the van der Waals force between nucleobases and the basal plane of MoS_2_ nanosheets, as well as helical wrapping to the MWCNT surface through п-stacking, and the fluorescence of the probe DNA is thus quenched. A double-stranded DNA (dsDNA) is formed when the perfect complementary target DNA (T1) is hybridized with P1. The fluorescence of P1 is retained well even with the presence of MoS_2_/MWCNT due to the weak binding of MoS_2_/MWCNT to dsDNA. As a result, P1 fluorescence intensity can offer a quantitative assessment of T1. However, when single-base mismatched (M1) and non-complementary DNA (N1) are used for hybridization, the strong fluorescence of P1 will be largely quenched in the presence of MoS_2_/MWCNT because of the formation of non-perfect dsDNA. Furthermore, the novel MoS_2_/MWCNT composite has a 3D hierarchical structure providing a large surface area for interacting with the DNA strand.

### 3.2. Fluorescence Quenching Ability of MoS_2_/MWCNT Nanocomposites

**Figure 2:**
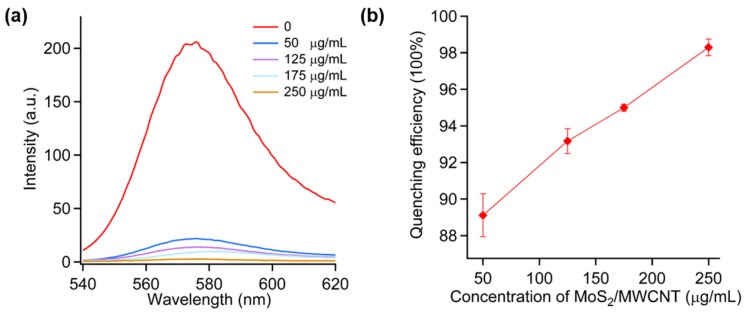
(**a**) Fluorescence spectra of P1 (100 nM) in the presence of various concentrations of MoS_2_/MWCNT (50, 125, 175, and 250 μg/mL); (**b**) Quenching efficiency of corresponding concentration of MoS_2_/MWCNT.

Figure S3 shows that a 1:5 ratio of MoS_2_/MWCNT has the highest quenching ability. This could be attributed to the formation of the hierarchical structure of MoS_2_ and MoS_2_ layers extruding from the sidewall of MWCNTs with the enhancement of the Mo/carbon ratio [26], which provides a larger interacting surface area for the DNA strand. The effect of the concentration of MoS_2_/MWCNT nanocomposites in the aqueous solution on the fluorescence quenching ability was investigated by measuring the fluorescence intensity in mixtures of 100 nM probe DNA and different concentrations of MoS_2_/MWCNT with the 1:5 ratio. Figure 2a depicts the fluorescence spectra of the 100 nM probe DNA quenched by different concentrations of MoS_2_/MWCNT. It is shown that the fluorescence intensity decreased with the increase of MoS_2_/MWCNT concentration. Figure 2b displays the quenching efficiency of MoS_2_/MWCNT that is defined as (*F*_0_ − *F*)/*F*_0_ *×* 100%, where *F* and *F*_0_ are the fluorescence intensities of the solutions at the wavelength of *λ* = 580 nm in the presence and absence of MoS_2_/MWCNT, respectively. It was found that the quenching efficiency is higher than 88%, even at a low concentration (50 μg/mL) of MoS_2_/MWCNT. Since the quenching efficiency of MoS_2_/MWCNT is very important for the proposed DNA sensor, we chose a 1:5 ratio and 250 μg/mL concentration reaching 98.3% quenching efficiency for the other assays in this work.

### 3.3. DNA Detection Sensitivity

**Figure 3:**
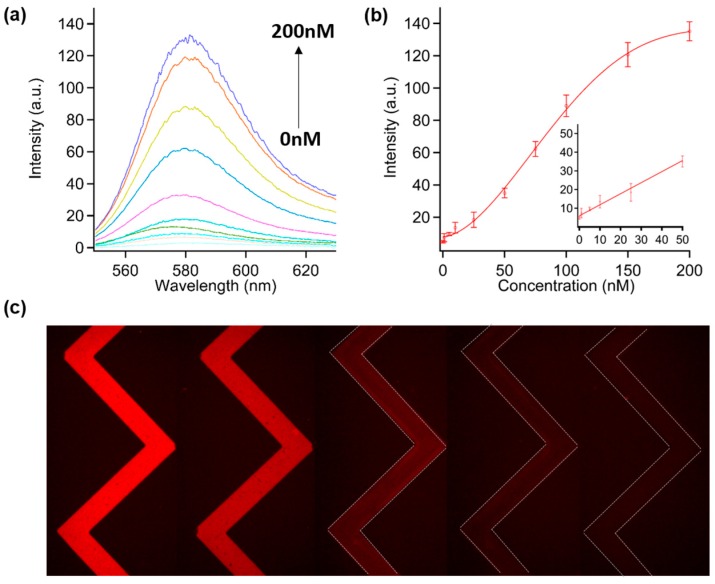
(**a**) Fluorescence spectra of P1 with a variety of T1 (0, 1, 5, 10, 25, 50, 75, 100, 150, and 200 nM) in the presence of MoS_2_/MWCNT (250 μg/mL); (**b**) Calibration curve for DNA detection; (**c**) Fluorescence images of P1T1 in the presence of various T1 concentrations (left to right; 150, 100, 75, 50 and 10 nM). The concentration of P1 is 100 nM.

The sensitivity of the proposed biosensor was investigated by changing the target DNA concentration. In a typical experiment, after 10 min incubation of P1 mixed with T1 at different concentrations (1–200 nM) at ambient temperature, MoS_2_/MWCNT nanocomposites were added to this hybridized P1T1 solution and mixed by using a vortex mixer and then added into microchannels. The detection time was less than 5 min and the DNA hybridization time was about 10 min. The fluorescence spectra of the above experiments with its corresponding derived calibration can be seen in Figure 3. The measurement of the spectra was performed at the end of the microchannels (Figure 3a). With the increase in T1 concentration, a larger amount of P1 was hybridized with T1 to form a dsDNA and resulted in higher fluorescence of P1 remained. It should be mentioned that, when the T1 concentration (150 and 200 nM) exceeded P1 (100 nM), there was still an increase in the fluorescence intensity. This might have arisen as a result of the fact that the redundant T1 has a stronger interaction with MoS_2_/MWCNT in comparison to hybridized P1T1 and could replace the P1T1 duplex adsorbed on MoS_2_/MWCNT; hence, a higher amount of P1T1 duplex remained in the solution. According to the derived calibration curve (Figure 3b), the detection limit of 1 nM with good linearity from 0 to 50 nM was achieved by this DNA biosensor. The linear range is wider than previous studies on MoS_2_-based fluorescence assays [13,30]. Notably, because the effective volume of DNA solution was less than 0.2 µL inside the microchannel, the presented microfluidic biosensor can detect the target DNA as low as 1 fmol, which is comparable to our previous work with pure MoS_2_ nanosheets [15] and much lower than other nanoprobe-based fluorescence methods in bulk solutions [13,30]. In addition, a table (Table S1) with a comprehensive comparison of various MoS_2_-based biosensors is provided in the supplementary information. Figure 3c demonstrates that the P1T1 fluorescence decreased along with the reduction of the T1 concentration, and ∼fmol of T1 produced a visible red color in the presence of MoS_2_/MWCNT.

### 3.4. DNA Detection Selectivity

**Figure 4:**
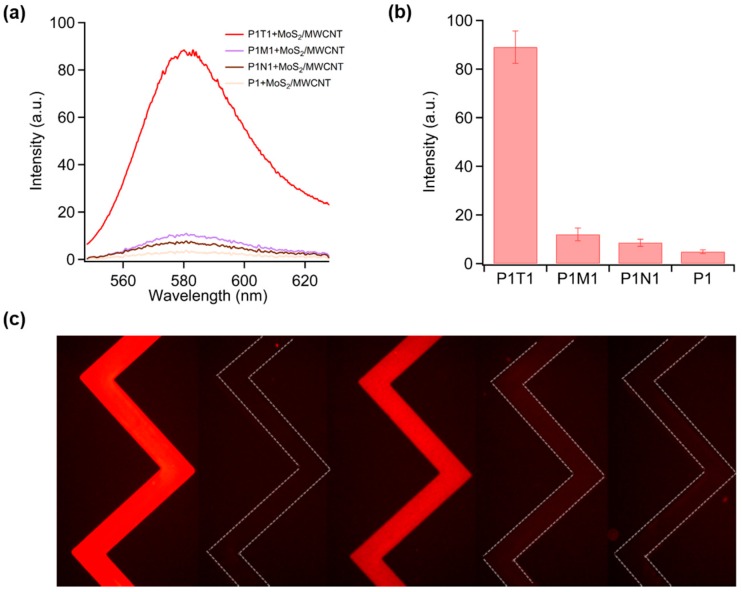
(**a**) Fluorescence spectra of 100 nM P1, P1T1, P1M1, and P1N1 in the presence of MoS_2_/MWCNT; (**b**) Selectivity of the MoS_2_/MWCNT-based target DNA (T1) sensor over single-base mismatched (M1) and non-complementary (N1) sequences; (**c**) Fluorescence images of P1, P1/(MoS_2_/MWCNT), P1T1/(MoS_2_/MWCNT), P1M1/(MoS_2_/MWCNT), and P1N1/(MoS_2_/MWCNT) from left to right. P1, T1, M1, and N1 have the same concentration of 100 nM.

The selectivity of the proposed methods was also examined by introducing the control experiments into the sensing system, including single-base mismatched ssDNA (M1) and non-complementary ssDNA (N1). As shown in Figure 4a,b, even at a very high concentration (100 nM), neither M1 nor N1 could produce a significant fluorescence increase. P1T1 showed a much stronger fluorescence than P1, P1M1, and P1N1 in the MoS_2_/MWCNT solution. It was also found that the fluorescence intensity of P1 was slightly lower than P1M1 and P1N1. This might be attributed to the redundant M1 or N1 that would compete with P1 and replace a small amount of the adsorbed P1 on MoS_2_/MWCNT; as a result, more P1 was maintained to produce slightly higher fluorescence intensity. Figure 4c illustrates that T1 induced a much stronger red color than M1 and N1 at the same concentration. The color of P1 appeared a bit weaker than that of P1 mixed with 100 nM M1 and N1 in the presence of MoS_2_/MWCNT, which is in good agreement with our fluorescence measurement (shown in Figure 4a,b). 

### 3.5. Stability of MoS_2_/MWCNT Aqueous Solution

**Figure 5:**
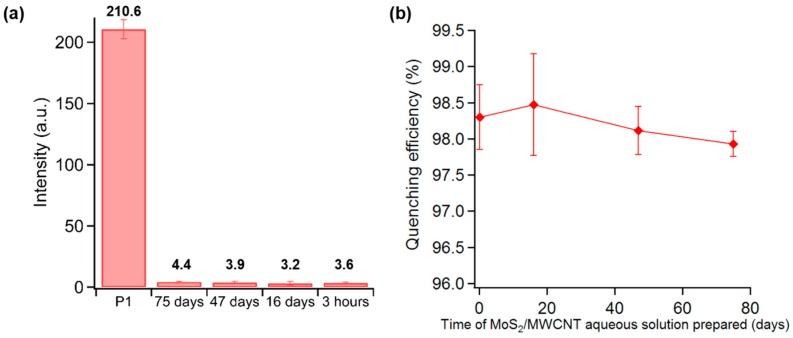
(**a**) Fluorescence intensity of 100 nM P1 at the emission wavelength of λ = 580 nm without and with MoS_2_/MWCNT prepared 75, 47, and 16 days prior, as well as 3 h prior; (**b**) Quenching efficiency of MoS_2_/MWCNT prepared 75, 47, and 16 days prior, as well as 3 h prior.

A significant advantage of the proposed biosensor stems from the high quenching stability of the MoS_2_/MWCNT aqueous solution compared with the pure MoS_2_ nanosheet aqueous solution. In a typical experiment, MoS_2_/MWCNT aqueous solutions prepared at different days (75, 47, and 16 days prior, as well as 3 h prior) were sonicated again for 10 min, mixed with 100 nM P1 by vortex mixer and then added into microchannels for fluorescence measurement. Figure 5a,b show that MoS_2_/MWCNT aqueous solution has a high stability of quenching ability, which can reach 98% even for MoS_2_/MWCNT aqueous solutions prepared 75 days prior. Therefore, prolonged storage of MoS_2_/MWCNT solutions for more than two months does not affect its quenching efficiency and stability. It is worth mentioning that these prepared MoS_2_/MWCNT solutions were kept in ambient temperature without any special ventilation or prevention from the average humidity of 80% in Singapore. This quenching stability will greatly reduce the time for sample preparation. Moreover, this simple, high-throughput, and homogeneous assay can be completed within a few minutes. Hence, the proposed method will greatly reduce time for large-scale DNA sensing studies.

## 4. Conclusions

We developed a novel 3D architectural MoS_2_/MWCNT nanocomposite-based microfluidic biosensor for the sensitive detection of DNA molecules. Several advantages are summarized as follows: (1) It can detect 1 nM DNA with 0–50 nM linear range; (2) Compared with other nanomaterials such as MoS_2_, the MoS_2_/MWCNT aqueous solution has an excellent quenching stability for storage more than 75 days without further processing, which is a great convenience for routine DNA tests; (3) The sample volume can be largely reduced with the use of microfluidic channels; hence, ~fmol DNA detection could be achieved with a visible color of fluorescence within a few minutes through the microfluidic assay. It offers a simple and rapid approach for high-throughput DNA analysis. The excellent quenching stability of the MoS_2_/MWCNT aqueous solution coupled with microfluidics can greatly simplify steps of DNA testing experiments and reduce time for large-scale DNA sensing studies, demonstrating that MoS_2_/MWCNTs can be a promising nanocomposite for versatile fluorometric biosensing.

## Figures and Tables

**Table 1:** Sequences of DNA oligonucleotides used in this study.

DNA Name	Sequence (5′–3′)
Probe ssDNA (P1)	5′-TAMRA-TGCGAACCAGGAATT-3′
Perfect complementary ssDNA (T1)	5′-AATTCCTGGTTCGCA-3′
Single-base mismatched ssDNA (M1)	5′-AATTCCTTGTTCGCA-3′
Non-complementary ssDNA (N1)	5′-CTGCAAGACCGGATT-3′

## Supplementary Materials

The following are available online at http://www.mdpi.com/1424-8220/16/11/1911/s1. Figure S1: Scanning electron microscopy (SEM) image of pristine multi-walled nanotubes (MWNTs). Figure S2: Channel geometry of the polydimethylsiloxane (PDMS) microfluidic device. The microchannel height is 40 µm and width is 100 µm. Figure S3: Fluorescence spectra of P1 (100 nM) in the presence of different ratios of MoS_2_/MWCNT (1:5, 1:10, and 1:20). The concentrations of the different MoS_2_/MWCNT ratios are all 150 µg/mL. Table S1: DNA detection performance of existing MoS_2_-based biosensors.
