# Supplementary material for: A Microfluidic DNA Sensor Based on Three-Dimensional (3D) Hierarchical MoS2/Carbon Nanotube Nanocomposites
Source: Sensors (Basel). 2016 Nov 14;16(11):1911. doi: 10.3390/s16111911 (PMC5134570; doi:10.3390/s16111911)
Supplement: Supplementary file 1 [file sensors-16-01911-s001.pdf]

Supplementary Materials: A Microfluidic DNA Sensor Based on Three-Dimensional (3D) Hierarchical MoS_2/Carbon Nanotube Nanocomposites

Dahou Yang, Mahnoush Tayebi, Yinxi Huang, Hui Ying Yang and Ye Ai

The scanning electron microscopy (SEM) of pristine multi-walled nanotubes (MWNTs) used in the experiments is shown in Figure S1. The outer diameter of the multi-walled carbon nanotubes (MWCNTs) range from 20 to 60 nm with a length of 5 to 15 μm. MoS_2 is extended the layered structure out of the cylindrical tubules, which is different from MoS_2 sheath/CNT-core nanoarchitecture where MoS_x layers are confined to the MWCNTs [1].

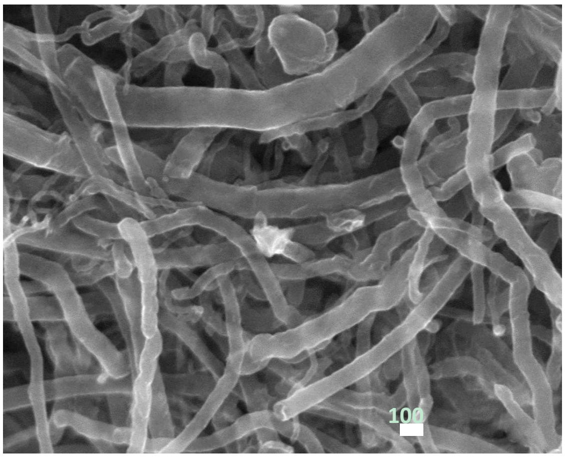

Figure S1. Scanning electron microscopy (SEM) image of pristine multi-walled nanotubes (MWNTs).

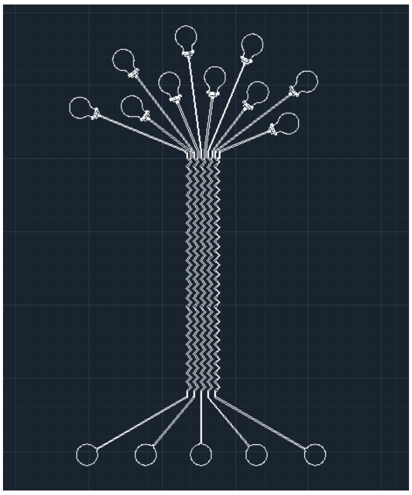

Figure S2. Channel geometry of the polydimethylsiloxane (PDMS) microfluidic device. The microchannel height is 40 μm and width is 100 μm.

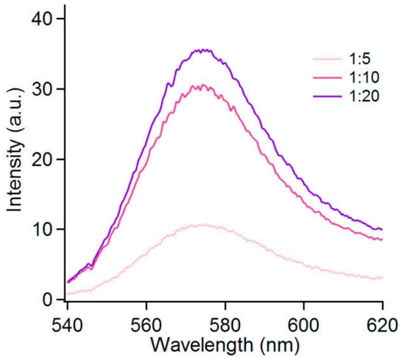

Figure S3. Fluorescence spectra of P1 (100 nM) in the presence of different ratios of MoS_2/MWCNT (1:5, 1:10, and 1:20). The concentrations of the different MoS_2/MWCNT ratios are all 150 μg/mL.

Table S1. DNA detection performance of existing MoS_2-based biosensors.

Sensor	Description	Linear Range (nM)	LOD (nM)	Reference
MoS_2 nanosheets	Using the fluorescence quenching ability of MoS_2 nanosheets toward the dye-labeled ssDNA was investigated in the zigzag-shaped microchannel	0–50	0.5	[2]
MoS_2 nanosheets	Using the fluorescence quenching ability of MoS_2 nanosheets toward the dye-labeled biomolecules was investigated in bulk samples	0–15	0.5	[3]
PL measurements of graphene/MoS_2 film	Photoluminescence mappings for the graphene/MoS_2 stack film immobilized with the probe DNA	0–0.000001	0.001×10^{-6}	[4]
MoS2-based method for DNA detection using hybridization chain reactions (HCRs)	MoS_2 is used to reduce the background signal and HCRs are employed to amplify the fluorescence emission	0–0.2	0.015	[5]
Few-layer MoS_2 nanosheets	Electrical detection of DNA by (2D) few-layer MoS_2 as a sensing-channel	-	0.00001	[6]
MoS_2 and WS_2 nanoflakes	Fluorescence detection of nucleic acids, based on a signal-on sensing approach	9.60–366	9.60	[7]
GOD/AuNPs/MoS2/MWCN Ts/GCE	For DNA sensing, cyclic voltammetry (CV) was carried out	0.00001–10	79×10^{-8}	[8]
CdS/MoS_2	Photoelectrochemical biosensor	10^{-6}–0.1	0.39×10^{-6}	[9]
MoS_2/MWCNT	Fluorescence quenching	0–50	1	This Work

Reference

1. Wang, Q.; Li, J. Facilitated lithium storage in MoS_2 overlayers supported on coaxial carbon nanotubes. *J. Phys. Chem. C* **2007**, *111*, 1675–1682.
2. Huang, Y.; Shi, Y.; Yang, H.Y.; Ai, Y. A novel single-layered MoS_2 nanosheet based microfluidic biosensor for ultrasensitive detection of DNA. *Nanoscale* **2015**, *7*, 2245–2249.
3. Zhu, C.; Zeng, Z.; Li, H.; Li, F.; Fan, C.; Zhang, H. Single-layer MoS_2-based nanoprobes for homogeneous detection of biomolecules. *J. Am. Chem. Soc.* **2013**, *135*, 5998–6001.
4. Chen, C.-H. Label-Free Detection of DNA Hybridization on MoS_2 Using Photoluminescence Measurements. In Proceedings of the 9th IEEE International Conference on Nano/Molecular Medicine & Engineering (NANOMED), Honolulu, HI, USA, 15–18 November 2015; pp. 196–199.
5. Huang, J.; Ye, L.; Gao, X.; Li, H.; Xu, J.; Li, Z. Molybdenum disulfide-based amplified fluorescence DNA detection using hybridization chain reactions. *J. Mater. Chem. B* **2015**, *3*, 2395–2401.
6. Lee, D.-W.; Lee, J.; Sohn, I.Y.; Kim, B.-Y.; Son, Y.M.; Bark, H.; Jung, J.; Choi, M.; Kim, T.H.; Lee, C. Field-effect transistor with a chemically synthesized MoS_2 sensing channel for label-free and highly sensitive electrical detection of DNA hybridization. *Nano Res.* **2015**, *8*, 2340–2350.
7. Loo, A.H.; Bonanni, A.; Pumera, M. Strong dependence of fluorescence quenching on the transition metal in layered transition metal dichalcogenide nanoflakes for nucleic acid detection. *Analyst* **2016**, *141*, 4654–4658.

8. Huang, K.-J.; Liu, Y.-J.; Wang, H.-B.; Wang, Y.-Y.; Liu, Y.-M. Sub-femtomolar DNA detection based on layered molybdenum disulfide/multi-walled carbon nanotube composites, au nanoparticle and enzyme multiple signal amplification. *Biosens. Bioelectron.* **2014**, *55*, 195–202.
9. Zang, Y.; Lei, J.; Hao, Q.; Ju, H. Cds/MoS_2 heterojunction-based photoelectrochemical DNA biosensor via enhanced chemiluminescence excitation. *Biosens. Bioelectron.* **2016**, *77*, 557–564.
